# Ultrasound-Assisted “Green” Extraction (UAE) of Antioxidant Compounds (Betalains and Phenolics) from *Opuntia stricta* var. *Dilenii*’s Fruits: Optimization and Biological Activities

**DOI:** 10.3390/antiox10111786

**Published:** 2021-11-08

**Authors:** Iván Gómez-López, Gloria Lobo-Rodrigo, María P. Portillo, M. Pilar Cano

**Affiliations:** 1 Laboratory of Phytochemistry and Plant Food Functionality, Biotechnology and Food Microbiology Department, Institute of Food Science Research (CIAL) (CSIC-UAM), Nicolás Cabrera 9, 28049 Madrid, Spain; ivan.gomez@ehu.eus; 2 Nutrition and Obesity Group, Department of Nutrition and Food Science, Faculty of Pharmacy, University of the Basque Country (UPV/EHU), Lucio Lascaray Research Center, 01006 Vitoria-Gasteiz, Spain; mariapuy.portillo@ehu.eus; 3 Department of Crop Production in Tropical and Subtropical Areas, Instituto Canario de Investigaciones Agrarias (ICIA), 38270 Tenerife, Spain; globo@icia.es; 4 BIOARABA Institute of Health, 01006 Vitoria-Gasteiz, Spain; 5 CIBERobn Physiopathology of Obesity and Nutrition, Institute of Health Carlos III (ISCIII), 01006 Vitoria-Gasteiz, Spain

**Keywords:** *Opuntia stricta* var. *Dillenii* whole fruits, ultrasound-assisted extraction, green solvents, betalains, phenolic compounds, antioxidant activity, anti-inflammatory activity

## Abstract

*Opuntia stricta* var. *Dillenii*’s prickly pears are an underutilized fruit with a high content of betalains and phenolic compounds that could bring potential health benefits for humans. The aim of this study is the optimization of the “green” extraction of betalains and phenolic compounds from *Opuntia stricta* var. *Dillenii*’s whole fruits by ultrasound-assisted extraction (UAE), using a response surface methodology (RSM) by a central composite design (CCD) in order to obtain extracts rich in betalains and phenolic compounds with proven biological activities. For UAE optimization, the extraction temperature (20–50 °C), the amplitude (20–50%) and the ethanol volume in extraction solvent (15–80%, *v*/*v*) were selected as independent variables. All combinations were conducted at 2, 5, 10, 20 and 30 min to determinate the time effect. The betalain and phenolic compound content in *Opuntia stricta* var. *Dillenii*’s whole fruits and UAE extracts were identified by HPLC-DAD-ESI/MS and HPLC-DAD-MS/QTOF and the antioxidant (ORAC method) and the anti-inflammatory (hyaluronidase inhibition method) in vitro biological activities also were determined. The most efficient extraction time was 5 min and the best UAE parameter combination was 50% amplitude, 15% ethanol in solvent (ethanol/water, 15/85, *v*/*v*) and 20 °C temperature, obtaining 10.06 ± 0.10 mg of total major betalains/g dry weight, 2.32 ± 0.08 mg of piscidic acid/g dry weight and 0.38 ± 0.00 mg of total major flavonoids/g dry weight. All applied UAE combinations significantly improved the in vitro bioactive activities (antioxidant and anti-inflammatory) of the *Opuntia stricta* var. *Dillenii*’s extracts compared to the bioactivities of the extracts obtained by standard homogenization processes.

## 1. Introduction

Prickly pear fruits (*Opuntia* spp. L Mill.) are originally from Mexico [1] but due to their capacity to grow in arid areas, they are also localized in different areas, such as Spain, Italy, India and Africa [2,3]. In Spain, they grow in different regions, such as the Canary Islands, Murcia, Almeria, among other places. However, the main production of prickly pears occurs in the Canary Islands [4]. *Opuntia* spp. has more than 200 species [2], being one of the most abundant genera within the *Cactaceae* family [5]. Nevertheless, there are wild species, such as *Opuntia stricta* var. *Dillenii,* that extensively grow in the Canary Islands, but they are not commercialized as fresh fruit and not widely consumed. This wild fruit is also an investigated *Opuntia* species.

The *Opuntia stricta* var. *Dillenii* fruit is like a small haw, and it is characterized by its dark purple color and by the huge among of seed that it has. In general, prickly pear fruits have a high nutritional value and, in addition, these fruits are a rich source of bioactive compounds such as betalains and phenolic compounds. The intensive purple color indicates that *Opuntia stricta* var. *Dillenii*’s fruits are a rich source of betacyanins (betalains with a purple color, a nitrogen-based dye), especially betanin. *Opuntia stricta* var. *Dillenii*’s prickly pear fruit is also rich in phenolic compounds, such as piscidic acid and flavonoids, mainly isorhammentin glucoxyl-rhamnosyl-pentoside (IG2) [6,7].

These bioactive compounds improve the appearance and flavor of the fruit but also bring potential health benefits for humans [8]. Pigments as betalains can improve the body’s redox balance and reduce lipid oxidation due to the antioxidant activity of scavenging the free radicals. This also displays health benefits as they are hepatoprotective and modulate gene expression [9,10,11]. Phenolic compounds also have health benefits because of their antioxidant, anti-inflammatory effects [12] and due to their role in adipogenesis, improving insulin resistance and reducing hepatic steatosis. Antunes-Ricardo et al. [13] reported this effect in obese rats attributed to the *Opuntia*’s isorhammentin flavonoids.

*Opuntia stricta* var. *Dillenii*’s prickly pear fruits are generally underutilized and their consumption is very low as fresh fruit. In Spain, only a few food factories use these *Opuntia* fruits as a raw material to produce jams and other derived products. Nowadays, consumer demand for healthy and functional foods is increasing. Gómez-López et al. [6] reported that the *Opuntia stricta* var. *Dillenii* whole fruits are an interesting starting material to obtain antioxidant compounds regarding not only their betalain and phenolic compound profile, but the stability and bioaccessibility of these bioactives during in vitro gastro-intestinal digestion. Consumers prefer foods that provide additional extra health benefits above the primary nutritional requirements. Shirazinia et al. [14] concluded that *Opuntia stricta* var. *Dillenii* needs more investigation to promote the benefits that it can give to the industries as the future of pharmapuncture [15]. This is the main reason why the interest in this fruit is increasing.

*Opuntia stricta* var. *Dillenii* fruits could be also a source of natural colorants and bioactive compounds with proven biological effects for food industrial applications. The extraction of *Opuntia* bioactives could be conducted by various technological processes. In the literature, many extraction processes can be found, such as homogenization, maceration and Soxhlet extraction. Over the last 50 years, non-conventional methods, such as ultrasounds (UAE), high hydrostatic pressures (HPAE) and microwaves (MAE), for extraction of bioactives have been developed. These methods are more environmentally friendly due to the decrease in solvent quantity necessary for extraction, the reduced operation time and the improved extraction yield and quality of extracts. Recent studies show that the ultrasound-assisted extraction method (UAE) could be one of the most efficient in comparison with other processes [16,17,18].

Ultrasound-assisted extraction (UAE) is based on the cavitation force formed by controlled amplitude waves. This force forms bubbles, which collapse in the plant tissue surface creating fissures through which the bioactive compounds are extracted by the solvent [19]. The use of this technology improves bioactive compound extraction by different mechanisms, such as material fragmentation, erosion, sonicapillary effects, sonoporation, etc. [20]. In addition, this innovative technology allows us to apply different extraction variables, such as temperature, time, solvent to solid ratio, different solvent compositions, amplitude and pulse time.

Several published studies have described the extraction of betalains and phenolic compounds from *Opuntia* cactus tissues. Melgar et al. [17] reported that the optimum recoveries of betalains and phenolics using UAE from *Opuntia engelmannii* fruits were a temperature of 30 °C, treatment time of 2.5 min, a solvent-to-solid ratio of 5 g/L and a solvent of 34.6% methanol in water (*v*/*v*). These authors obtained a maximum of 201.6 mg/g of dry extracts of betalains, 13.9 mg/g of dry extract of phenolic acids and 2.4 mg/g dry extract of flavonoids, but they employed methanol as a solvent, which is not a green solvent. Karatuanithi et al. [16] in their study about UAE extracted phenolic compounds from *Opuntia ficus-indica* obtained as a maximum 5.95 mg gallic acid eq./g dry weight (total phenolic compound) and 9.79 mg rutin eq./g dry weight (total flavonoids), with a temperature of 40 °C, an extraction time of 17 min and a solid-solvent ratio of 1:24 g/mL as the optimum recovery conditions. In both of these studies the ultrasound-assisted extraction (UAE) was conducted with mixtures of methanol/water (*v*/*v*) as extraction solvents, which is supposed to be a non-environmentally friendly process.

In the present study, we used an underutilized *Opuntia* wild variety, such as *Opuntia stricta* var. *Dillenii* from the Canary Islands (Spain), as the starting material to obtain extracts rich in betalains and phenolic compounds with higher antioxidant and anti-inflammatory activities and an almost unaltered profile composition by UAE, using a green solvent composed of mixtures of ethanol/water. The amplitude (%) of the ultrasound, the extraction temperature (°C), the solvent composition in ethanol/water (%, *v*/*v*) and the extraction time (min) were studied as UAE independent variables. The range of the process variables were selected in order to obtain a better extraction yield with higher bioactive content, and with better antioxidant and anti-inflammatory bioactivities. The UAE process must be environmentally friendly (by a reduction in the extraction time, by the use of environmentally damaged solvents and by consuming less energy).

## 2. Materials and Methods

### 2.1. Solvents, Reagents and Standards

Ultra-pure MiliQ water was obtained from a Milipak^®^ Express 40 system (Merk-Milipore, Dormstadt, Germany). Ethanol (99.97%) and methanol (99.8% LC-MS) were purchased from VWR International (Barcelona, Spain). The betanin was purified by a Sephadex L20 resin from a commercial beetroot concentrate extract. Using the purified betanin, betaxanthins were semi-synthesized based on methods reported by García-Cayuela et al. [3]. Phyllocactin was isolated from *Myrtillocactus geometrizans* fruits (cactus berry, garambullo), applying a semi-preparative high-performance liquid chromatography (HPLC) method reported by Montiel-Sánchez et al. [21]. Piscidic acid was purified from prickly pear peels also using a semi-preparative high-performance liquid chromatography (HPLC) reported by García-Cayuela et al. [3]. Purified standards of flavonoids as the different isorhamnetin glycosides were provided by Dr. Serna-Saldivar’s laboratory (Biotechnology center FEMSA of Instituto Tecnologico de Monterrey, Monterrey, Mexico). Other phenolic compounds, such as gallic acid, ferulic acid, protocatechuic acid, p-hydroxybenzoic acid, quinic acid, ellagic acid, p-coumaric acid, quercetin, myricetin, rutin and kaempferol standards, were purchased from Sigma-Aldrich (St. Louis, MO, USA).

For in vitro assays of biological activities, sodium chloride, sodium acetate, sodium hydroxide and sodium di-hydrogen phosphate were acquired from Panreac Quimica SLU (Barcelona, Spain). CTAB (hexadecyltrimethyl-ammonium bromide), hyaluronidase enzyme, APPH (APPH (2, 2′-azobis (2-amidino-propane) dihydrochloride) and Trolox were purchased by Sigma-Aldrich (St. Louis, MO, USA). Potassium hydrogen phosphate was obtained from Merck KGaA (Darmstadt, Germany). Sodium hyaluronidase was supplied by Acros organics (Fairlawn, NJ, USA).

### 2.2. Plant Material

*Opuntia stricta* var. *Dillenii*’s wild fruits were harvested in September of 2020 from Tinajo, Lanzarote, Canary Islands (Spain; 29°3′ N, 13°4′ W; 209 m above sea). Whole fruits were selected according to the size and skin color of the fruits, discarding damaged fruits. Then, twenty fruits were washed and air dried and they were used to perform physicochemical analysis in order to establish the fruit maturity. All other whole fruits were cut in small pieces of 1 cm × 1 cm and were immediately frozen with liquid nitrogen and freeze dried for 5 days at −45 °C and 1.3 × 10^−3^ MPa (Lyobeta 15, Azbil telstar, S.L., Terrasa, Spain). Freeze-dried prickly pear whole fruits were pulverized in a knife mill (Grindomix GM200, Retsch, Germany) to a small particle size (<2 mm) and sieved to remove the seeds. Samples were stored at −24 °C in vacuum-sealed plastic bags until analysis and UAE extraction assays.

### 2.3. Physicochemical Analysis

All physicochemical analysis was conducted as reported by Gómez-López et al. [6]. pH and soluble solids were measured from fresh pulp fruit puree. Titratable acidity was carried out by neutralization of the fruit juice obtained from fresh fruit pulp puree with 0.1 N sodium hydroxide until reaching a 8.1 pH value. Wet basis moisture was determined by drying the fresh pulp up to a constant weight by microwave oven. Colors of peel and pulp were determined by the CIELAB system with a Konica Minolta CM-3500D colorimeter (Osaka, Japan). Table S1, Supplementary Material, shows all these physicochemical characteristics of the fresh *Opuntia stricta* var. *Dillenii*’s fruits.

### 2.4. Analysis of Betalain and Phenolic Compounds of Opuntia stricta var. Dillenii Whole Fruits

#### 2.4.1. Extraction of Betalains and Phenolic Compounds for HPLC Analysis

The extraction of betalains and phenolic compounds for HPLC analysis of the *Opuntia* material (whole fruits) was performed following the procedure reported by Gómez-López et al. [6]. This method is the conventional procedure to obtain extracts without the use of ultrasound. Briefly, 0.5 g of the freeze-dried *Opuntia* whole fruit freeze-dried sample was extracted by homogenization with 5 mL methanol/water (50/50, *v*/*v*) for 2 min and 25,000 rpm using Ultraturax (T-25 Digital, IKA works inc., Staufen Germany) with an external sample cooling to avoid an increase in the temperature. Later, the mixture was centrifuged for 10 min at 10,000 rpm and at 4 °C. The supernatant was separated, and solid residues were re-extracted two more times using 3 mL methanol/water (50/50, *v*/*v*), and one last time with 3 mL of 100% methanol. Obtained supernatants were rotoevaporated (Buchi, Flawil, Switzerland) at 25 °C to minimum volume and then made up to 5 mL with MilliQ water. A total of 1 mL of the extract was filtered through a 0.45 µm membrane to be analyzed by HPLC. The rest of the volume of the extract was immediately frozen and stored at −24 °C until the antioxidant and anti-inflammatory bioactivity assays. the entire extraction procedure was carried out under soft light, avoiding exposure to oxygen.

#### 2.4.2. HPLC Analysis of Betalains and Phenolic Compounds

Betalains and phenolic compounds were simultaneously determined according to the method reported by our research group [6]. A 1200 Series Agilent HPLC System (Agilent technologies, Santa Clara, CA, USA) with a C18, reverse column (Zorbax SB-C18, 250 × 4.6 nm i.d., S-5 µm; Agilent) at 25 °C was employed. Ultrapure water with %1 formic acid (*v*/*v*) and methanol with 1% formic acid were used as Phase A and B in a gradient during 70 min. An injection volume of 20 µL and a flow rate of 0.8 mL/min were utilized. A UV-Visible photodiode array detector was used to quantify the bioactive compounds at wavelengths of 280 nm (phenolic acids), 370 nm (flavonoids), 480 nm (betaxanthins) and 535 nm (betacyanins). To confirm the chemical composition of these bioactives, an HPLC-DAD-MS/QTOF analysis was also conducted using an electrospray ionization (ESI) mass spectrometry detector (LCMS SQ 6120, Agilent technologies, Santa Clara, CA, USA). Furthermore, to complete the characterization of bioactive compounds, mass spectrometry analyses were performed using maXis II LC-QTOF equipment (Bruker Daltonics, Bremen, Germany) with an ESI source and the selfsame chromatographic shape. All chromatographic procedures and mass spectroscopy conditions were previously reported by Gómez-López et al. [6].

### 2.5. Optimization of Betalain and Phenolic Compound Ultrasound-Assisted Extraction (UAE)

#### 2.5.1. Experimental Design

To optimize the ultrasound-assisted extraction (UAE) of bioactive compounds from *Opuntia stricta* var. *Dillenii*’s whole fruits, a central composite design (CCD) with response surface methodology (RSM) was carried out. Design-Expert software, version 11 (State ease Inc., Minneapolis, MN, USA) was used to design 16 combinations of the proposed variables, which includes 2 central points and 4 factorial and 4 axial points. In order to minimize the effect of unexpected variability, the experimental runs were randomized, and two independent replications were made for each combination.

The independent variables (temperature (20–50 °C); amplitude (20–50%) and ethanol volume in solvent (15–80% (*v*/*v*)), run number, levels and CCD combinations are shown in Table 1.

The pulse duration (on:off, seg:seg), solvent-to-solid ratio (*w/v*) and extraction time (min) were maintained constant for all CCD combination assays. A ratio of 1:1 (on:off, seg:seg) was applied based on the previous studies of Chemat et al. [20] and Kaderides et al. [22]. Solvent-to-solid ratio was kept at 0.2 g/L based on a previous study reported by Melgar et al. [23], and all CCD combinations were conducted at five (5) different extraction times: 2, 5, 10, 20 and 30 min.

#### 2.5.2. Ultrasound-Assisted Extraction (UAE)

Ultrasound-assisted extraction (UAE) assays were carried out using ultrasound equipment (Digital sonifier, Branson Ultrasonics corporation, Danbury, CT, USA) with a 13 mm diameter ultrasound-probe (Biogen Cientific S.L, Madrid, Spain). Assays were carried out mixing 0.5 g of the freeze-dried *Opuntia stricta* var. *Dillenii* whole fruit sample with 25 mL of each solvent mixture and were processed according to the above-described CCD design (Table 1), where different levels of temperature (20–50 °C), amplitude (20–50%) and ethanol percentage in the solvent (ethanol/water (15–80%), (*v*/*v*)) were combined. Temperature was kept at the prefixed value using an external equipment water bath (Memmert GmbH + Co. KG, Germany). After the UAE assay, the extraction mixtures were centrifuged at 10,000× *g* at 4 °C. Supernatants were dried (Buchi, Flawil, Switzerland) at 25 °C, avoiding the light and oxygen to the minimum volume and later, they were immediately lyophilized for 5 days at −45 °C and 1.3 × 10^−3^ MPa (Lyobeta 15, Azbil telstar, S.L., Terrasa, Spain) and stored at −24 °C until the HPLC analysis of bioactives and in vitro bioactivity analysis. Freeze-dried UAE extracts were dissolved in MilliQ water up to 10 mL and filtered through a 0.45 µm membrane before HPLC analysis.

#### 2.5.3. Statistical Analysis and UAE Process Modelling

The variable response yields, Y1 (total major betalains (mg/g dry weight), resulting in the sum of the most abundant identified betalains in *Opuntia stricta* var. *Dillenii* whole fruit extracts, such as betanin, isobetanin, 2′-O-apiosyl-4-O-phyllocactin, 5″-O-E-sinapoyl-2′-apyosil-phyllocactin and neobetanin), Y2 (piscidic acid (mg/g dry weight), the most abundant phenolic acid in *Opuntia stricta* var. *Dillenii* fruits), Y3 (total major flavonoids (mg/g dry weight), as the sum of the most abundant identify flavonoids: isorhamnetin glucoxyl-rhamnosyl-pentoside (IG2), Quercetin-3-O-rhamnosyl-rutinoside (QG3) and Quercetin glycoside (QG2)—Quercetin hexose pentoside), Y4 (extraction yield of major betalains (%)), Y5 (extraction yield of major phenolic acid), Y6 (extraction yield of major flavonoids (%)), Y7 (antioxidant activity (ORAC method) (µmol trolox eq./g dry weight) and Y8 (anti-inflammatory activity (% of hyaluronidase inhibition)) were selected as the variables to optimize the ultrasound-assisted extraction process. Fitting procedures, coefficient estimates and polynomial model equations were conducted using the Design-Expert^®^ software. The extraction yield was calculated with the following equation (Equation (1)):

Y (Yield %) = Content of bioactives in Ultrasound extract^a^ (mg/g dry weight)/Content of bioactives in Control extract^b^ (mg/g dry weight) × 100 (1)

^a^ Ultrasound extracts; ^b^ Extract obtained by the conventional method without ultrasound (described in Section 2)

### 2.6. Analysis of the Opuntia stricta var. Dillenii Extracts Obtained by UAE

#### 2.6.1. HPLC Analysis of Betalain and Phenolic Compounds of UAE *Opuntia stricta* var. *Dillenii* Whole Fruit Extracts

Extracts obtained by UAE were first liophylized and stored at stored at −24 °C until HPLC analysis. Liophylized extracts were dissolved to 10 mL with MilliQ water. After, 1 mL of this solution was filtered (0.45 µm nylon filter (E0032, Análisis Vínicos SL, Tomelloso, Spain) and immediately analyzed by HPLC. The HPLC procedure was previously reported by Gómez-López et al. [6].

#### 2.6.2. Determination of the In Vitro Antioxidant Activity of *Opuntia stricta* var. *Dillenii* Whole Fruit UAE Extracts

Antioxidant activity was determinate by ORAC (oxygen radical absorbance capacity) assay described by Gómez-Maqueo et al. [24] for *Opuntia* fruits. The antioxidant capacity was measured by fluorescence degradation according to the antioxidant capacity of the target compounds. The assay was conducted in an automated plate fluorimeter reader (Bio-Tek Instruments Inc., Winooski, VT, USA) in a 96-well microplate. Extracts were diluted in PBS (phosphate-buffered saline) at 0.075 M and pH 7.4. In the microplate, 20 µL of previous diluted extracts and 120 µL of 11.7 µM fluorescein disodium (as substrate) were added and incubated for 10 min at 37 °C. After, 60 µL of 0.153 M APPH (2, 2′-azobis(2-amidino-propane) dihydrochloride) was added to generate peroxyl radicals. In the plate reader, 91 measures were recorded over 91 min (1 per minute) under the following conditions: excitation wave at 485 nm and emission wave at 520 nm. The antioxidant activity was calculated using a Trolox curve and elaborated with a range between 2 and 10 mM. Results were expressed as µmol Trolox equivalents/g dry weight.

#### 2.6.3. Determination of the In Vitro Anti-Inflammatory Activity of *Opuntia stricta* var. *Dillenii* Whole Fruit UAE Extracts

Anti-inflammatory activity was determined by hyaluronidase inhibition following the procedure described by Gómez-Maqueo et al. [24] for *Opuntia* fruits. A total of 15 µL of extract or standard samples were placed in a 2 mL vial along with 147 µL acetate buffer (0.2 M sodium acetic acid at pH 6 with 0.15 M NaCl). Afterwards, 120 µL of sodium hyaluronidase (0.5 mg/mL) was added and mixed for 30 s. Then, 18 µL of hyaluronidase (1 mg/mL) was added to the reaction mixture. In order to carry out the reaction, reaction mixtures were incubated at 37 °C for 15 min. The reaction was stopped by the addition of 1.2 mL of 2.3% CTAB (hexadecyltrimethyl-ammonium bromide) (*w/v*) in 2% NaOH (at pH 12). Other incubation assays were carried out at room temperature (25 °C) for 10 min. Then, the samples and blanks were analyzed by reading the absorbance at 400 nm in a spectrophotometer reader (SmartSpect Plus, BIO-RAD, Hercules, CA, USA). A sample blank was used to subtract any possible betalain interference at 400 nm. Results were expressed as hyaluronidase inhibition percentage (%).

### 2.7. Statistical Analysis

All of the data were analyzed by SPSS Statistics software 26.0 for Windows (IBM corp., Armonk, NY, USA). Significant differences (*p* < 0.05) between independent variables were calculated by one-way analysis of variance (ANOVA) using post hoc Tukey’s-b test. All results were expressed as mean ± standard deviation of at least three independent determinations (*n* = 3) for analysis of antioxidant compounds and biological activities.

## 3. Results and Discussion

### 3.1. Characterization and In Vitro Bioactive Properties of the Opuntia stricta var. Dillenii Whole Fruits (Starting Material) and UAE Extracts

The physicochemical characteristics and total bioactive compound contents of *Opuntia stricta* var. *Dillenii* whole fruits and the in vitro biological activities (antioxidant and anti-inflammatory activities) of *Opuntia stricta* var. *Dillenii* whole fruits are shown in Table S1. Additionally, Tables S2 and S3 (Supplementary Material) show the identification and content of the individual betalains and phenolic compounds of the starting material *O. Dillenii* whole fruits. These data have been previously reported by Gómez-López et al. [6] and were used to calculate the yield of the UAE assays for different bioactive compounds.

*Opuntia stricta* var. *Dillenii* whole fruits, the starting material for the UAE assays, had a pH of 3.32 ± 0.02, a titratable acidity of 1.58 ± 0.10 g citric acid/100 g fresh weight, and a soluble solid value of 12.63 ± 0.85°Brix. As other authors reported, these *Opuntia* fruits have a higher titratable acidity than such other *Opuntia* varieties as orange Colorada (pH 6.1 ± 0.2), red Fresa (pH 6.1 ± 0.00), white Blanco Buenavista (pH 6.6 ± 0.01) and white Blanco Fasnia (pH 6.2 ± 0.01) varieties from *Opuntia ficus-indica* L. Mill. [7,10]. The intense purple colored *Opuntia stricta* var. *Dillenii* fruit pulp was analyzed by CIELAB colorimeter parameters (30.82 ± 1.29 L*, 4.58 ± 0.34 a*, (−7.36) ± 0.43 b*). These values are very similar to those reported by Gómez-López et al. [6] for *Opuntia* var. *stricta Dillenii* fruit tissues and by-products of their industrialization.

The identification of the individual bioactive compounds was conducted according to the retention time, *UV/Vis*, and mass spectra data, based on a recently published study [6]. In Table S2 (Supplementary Material), the characterization of the major bioactive compounds identified in the whole fruits of *Opuntia stricta* var. *Dillenii* is shown, and in Table S3, the content in each compound is shown. Figure S1 (Supplementary Material) shows the HPLC-DAD chromatograms obtained at 280 nm, 370 nm, 480 nm and 535 nm of the whole fruit extracts with the identification of the most abundant bioactive compounds (betalains and phenolic compounds). In *Opuntia stricta* var. *Dillenii* whole fruits, Betanin (peak 2) and 5″-O-E-sinapoyl-2′-apyosil-phyllocactin (peak 5) were the most abundant betalains, with 2.74 ± 0.02 g/g dry weight and 2.77 ± 0.01 mg/g dry weight, respectively. *Opuntia stricta* var. *Dillenii* whole fruits were also a source of Isobetanin (peak 3) with 1.68 ± 0.01 mg/g dry weight, Neobetanin (peak 6) with 1.64 ± 0.00 mg/g dry weight and 2′-O-apiosyl-4-O-phyllocactin (Peak 4) with 1.22 ± 0.01 mg/g dry weight.

In the present study, the starting material, *O. Dillenii* whole fruit, had a 10.05 mg/g dry weight of total betalain content, calculated as the sum of the content of the individual betalain contents analyzed by HPLC-DAD. This material is the best one due to its high content of betalains with respect to other *O. Dillenii* fruit tissues (peel or pulp) and by-products [6]. *Opuntia stricta* var. *Dillenii*’s prickly fruits are also rich in phenolic compounds, such as piscidic acid (peak 1), with 0.93 ± 0.00 mg/g dry weight, these being the most abundant in these phenolic chemical families [6,7]. In addition, these fruits are also a rich source of flavonoids, such as isorhammentin glucoxyl-rhamnosyl-pentoside (IG2) (peak 9) with 0.26 ± 0.01 mg/g dry weight, Quercetin-3-O-rhamnosyl-rutinoside (QG3) (peak 7) with 0.02 ± 0.01 mg/g dry weight and Quercetin glycoside (QG2)—Quercetin hexose pentoside (peak 8) with 0.05 ± 0.00 mg/g dry weight. The sum of the major flavonoids was 0.33 ± 0.00 mg/g dry weight (Table S3, Supplementary Material).

With respect to the in vitro biological properties, *Opuntia stricta* var. *Dillenii* whole fruits showed an antioxidant activity value of 151.81 ± 1.86 µmol trolox eq./g dry weight and an anti-inflammatory activity of 22.51 ± 2.52% by hyaluronidase inhibition (Table S1). Gómez-Maqueo et al. [25] reported that *O. ficus indica* L. Mill. prickly pears from Sanguinos and Pelota varieties have antioxidant activities of 81.4 ± 11.5 µmol trolox eq./g dry weight and 56.9 ± 3.2 µmol trolox eq./g dry weight, respectively. The starting material of this study (*Opuntia stircta* var. *Dillenii* whole fruit) had almost twice the antioxidant activity reported for other *Opuntia* spp., such as *O. ficus indica* L. Mill. Regarding the anti-inflammatory activity determined by hyaluronidase inhibition (%), the *O. ficus indica* L. Mill. Pelota and Sanguinos fruit varieties have slightly higher activities, 35.8 ± 2.5% and 32.4 ± 0.8%, respectively, than those observed in the present study for *Opuntia stricta* var. *Dillenii* whole fruits.

Figure S2 (Supplementary Material) shows the HPLC-DAD obtained at 280 nm, 370 nm, 480 nm and 535 nm of the obtained extract from ultrasound-assisted extraction (UAE) at 50% amplitude, 15% ethanol in solvent (ethanol/water, 15/85, *v*/*v*) and 20 °C temperature (parameter combination, run 10) with the identification of the most abundant bioactive compounds (betalains and phenolic compounds). The chromatographic profile of betalains and phenolics obtained with the UAE process and the obtained one with conventional extraction (see Section 2) did not show any significant differences (Figure S1). However, the content of the different identified compounds was different among UAE extracts (Table 2) and conventional extracts (controls) (Table S3). The most abundant betalains in UAE extracts were betanin (peak 2), isobetanin (peak 3), 5″-O-E-sinapoyl-2′-apyosil-phyllocactin (peak 5) and neobetanine (Peak 6) (Figure S2). With respect to phenolic compounds, piscidic acid (peak 1) and isorhammentin glucoxyl-rhamnosyl-pentoside (IG2) (peak 9) were the most abundant compounds in UAE extracts obtained at run 10 (Figure S2).

### 3.2. Optimization of Ultrasound-Assisted Extraction (UAE) of Bioactive Compounds from Opuntia stricta var. Dillenii Whole Fruits

#### 3.2.1. Effect of Time in Ultrasound-Assisted Extraction (UAE)

**Figure 1:**
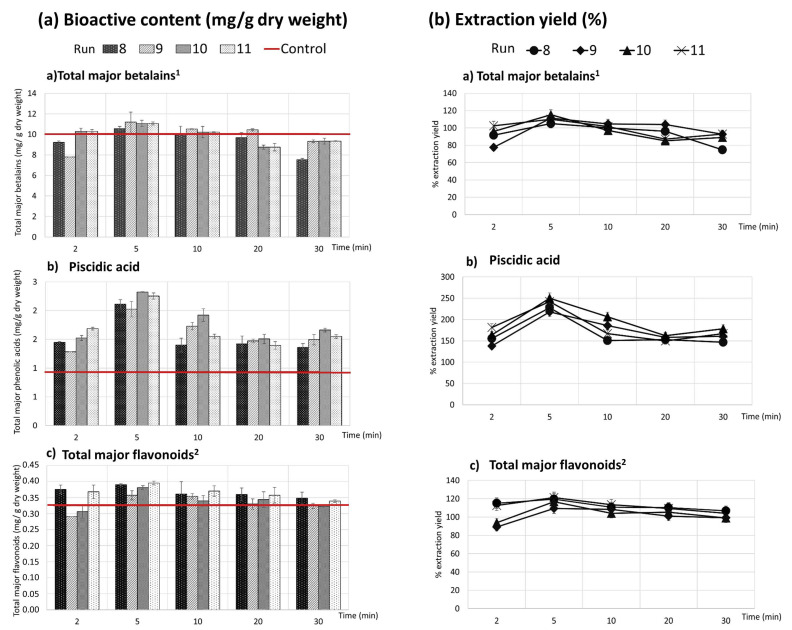
Process time effect on the content of major bioactive compounds and extraction yield of total major betalains, piscidic acid and flavonoids: ^1^ the sum of most abundant betalains: betanin, isobetanin, 2′-O-apiosyl-4-O-phyllocactin, 5″-O-E-sinapoyl-2′-apyosil-phyllcactin and neobetanin, ^2^ the sum of most abundant flavonoids: Isorhamnetin glucoxyl-rhamnosyl-pentoside (IG2), Quercetin-3-O-rhamnosyl-rutinoside (QG3) and Quercetin glycoside (QG2)—Quercetin hexose pentoside, obtained after selected UAE treatments: run 8 (35% amplitude, 47,5% ethanol in solvent (*v*/*v*), 60 °C temperature); run 9 (20% amplitude, 15% ethanol in solvent (*v*/*v*); 50 °C temperature); run 10 (50% amplitude, 15% ethanol in solvent (*v*/*v*); 20 °C temperature); and run 11 (35% amplitude, 47,5% ethanol in solvent (*v*/*v*); 35 °C temperature).

Figure 1 shows the effect of the extraction time (min) in the bioactive content (mg/g dry weight) and in the extraction yield (%) at different UAE treatment combinations (CCD described design in Table 1). In Figure 1, the following assays are represented: run 8 (35% amplitude, 47,5% ethanol in solvent (*v*/*v*), 60 °C temperature); run 9 (20% amplitude, 15% ethanol in solvent (*v*/*v*); 50 °C temperature); run 10 (50% amplitude, 15% ethanol in solvent (*v*/*v*); 20 °C temperature); and run 11 (35% amplitude, 47,5% ethanol in solvent (*v*/*v*); 35 °C temperature). These runs have been selected because they are the most representative of the effect of the process time and because the obtained extracts showed the highest concentration of bioactive compounds.

Extraction yield (%) and the bioactive content (mg/g dry weight) were dependent on time in all experiments (Table 2). Treatment time (2, 5, 10, 20, 30 min) was selected based on previously published studies about the ultrasound-assisted extraction of betalains from *Amaranthus caudatus* L. flowers [19,21]. Attending to the total major betalains (as a sum of the individual contents of betanin, isobetanin, 2′-O-apiosyl-4-O-phyllocactin, 5″-O-E- sinapoyl-2′-apyosil- phyllocactin and neobetanin) and to the total major flavonoids (as a sum of Isorhamnetin glucoxyl-rhamnosyl-pentoside (IG2), Quercetin-3-O-rhamnosyl- rutinoside (QG3) and Quercetin glycoside (QG2)—Quercetin hexose pentoside), the extraction yield increased during the first 5 min. However, after this time, extraction yield decreased. Run 10 conditions (50% amplitude, 15% ethanol in solvent (*v*/*v*)) at 5 min) extracted a total major betalain content of 11.59 ± 0.31 mg/g dry weight and 0.38 ± 0.00 mg/g dry weight of total major flavonoids. Comparing the run 10 conducted at 5 min to the assay conducted for 2 min, the extraction yield increased to 20.28 ± 1.07% for total major betalains and to 19.75 ± 1.69% for total major flavonoids. However, at 10 min of the UAE process time, the recovery decreased to 15.83 ± 2.57% for total major betalains and to 12.32 ± 1.04% for major flavonoids (Figure 1) compared with that conducted at 5 min (Figure 1).

In the case of the major phenolic acid detected in whole fruit UAE extracts (piscidic acid), Figure 1 also shows how its yield and content increased, but from this time forward, they significantly decreased. UAE assay carried out with run 10 conditions (50% amplitude, 15% ethanol in solvent (*v*/*v*)) at 5 min) produced extracts with a piscidic acid content of 2.32 ± 0.08 mg/g dry weight, Table 2. Comparing the run 10 conducted at 5 min to the 2 min treatment time, the extraction yield increased to 86.03% at 5 min; however, at a time of 10 min, the yield decreased to 43.28%.

In a previous study, Melgar et al. [17] compared microwave and ultrasound-assisted extractions of betalains from *Opuntia engelmannii* cultivar (cv.) peel using methanol/water as the extraction solvent and reported that the optimum time to extract betalains using ultrasounds was 2.5 min. Other studies of betalain ultrasound-assisted extraction from *Chenopodium quinoa willd* also reported that short ultrasound extraction process times were more effective in extracting betalains than longer times [26]. In the present study, short times also increase the extraction yield of the betalains using mixtures of ethanol/water as solvents (Figure 1). Maran et al. [26] in a study about the ultrasound-assisted extraction (UAE) of phenolic compounds using an ultrasonic bath from *Nephelium lappaceum* L. fruit peel concluded that the extraction yield increased and was maintained when the process time was in the range of 10–20 min. After that time, the extraction yield decreased slowly. In the present study, a direct probe was used in the UAE process (see Section 2), which could be the reason why the most effective extraction time was significantly shorter (5 min). However, in both cases, when an ultrasound bath or ultrasound direct prove were used, the extraction yield trend was similar, showing that longer times produced lower extraction yields.

Long-term UAE treatment produced more cavitation bubbles in the extraction mixture (solid + solvent) and the collapse of bubbles near the surfaces was also associated with the produced turbulences that might change the surfaces and could damage the bioactive compounds present in the extracts [27]. This might induce the degradation of betalains and phenolic compounds, so in the present study, the UAE treatments conducted at 5 min produced the most efficient extraction yields (Figure 1). For this fact, this process time was selected as the optimum time for the UAE extraction of betalains and phenolic compounds from *Opuntia stricta* var. *Dillenii* whole fruits.

#### 3.2.2. Experimental Data for Process Optimization

Table 2 shows the experimental results of the UAE assays conducted at 5 min: extraction yield (%), bioactive content in UAE extracts and the in vitro bioactivities of the UAE extracts. In the present study, all possible ranges of ethanol percentage in the extraction solvent (0–100%, *v*/*v*) were assayed. Melgar et al. [17] concluded that with less methanol volume in the solvent, a better extraction yield of bioactive compounds was obtained due to the solvent mixture polarity. In the present UAE study, ethanol was used as a green solvent instead of methanol, but all the obtained data indicated that with a lower ethanol volume, a better extraction yield was achieved, with more total betalains and phenolic compounds (mg/g dry weight) in the extracts. The assayed temperature range in the present UAE study was selected based on previous studies of betalain ultrasound-assisted extraction from quinoa and on reported studies about betalains’ thermostability [25,28]. Temperature of the UAE process plays an important role in bioactive compound extraction, as Chemat et al. [20] reported in a review about the knowledge of ultrasound-assisted extraction (UAE) technology in food ingredient development. In addition, the ultrasonic amplitude levels assayed in the present study were selected according to a reported work of Kaderides et al. [22] which described the study of UAE extraction of bioactive compounds from pomegranate peels.

##### Betalains

The obtained target responses (extraction yield (%) and the content (mg/g dry weight)) of total major betalains were from 0.43 to 115.9% and from 0.04 to 11.59 mg/g dry weight, respectively. The lowest values in two target responses were obtained at run 3 (amplitude 20%, ethanol 100% in solvent and 35 °C), exactly 0.43 ± 0.08% for the extraction yield of betalains and 0.04 ± 0.01 mg/g dry weight. In this combination (run 3), the color of the extracts was very pale, and this could indicate that a very low quantity of betalains was present. The highest extraction yields and content of betalains were obtained (without statistically significant differences, *p >* 0.05) at run 9 (amplitude 20%, 15% ethanol in solvent (*v*/*v*) and 60 °C), showing a 111.34 ± 9.72% extraction yield and 11.59 ± 0.11 mg/g dry weight of total major betalains, Table 2. Additionally, run 10 (amplitude 50%, 15% ethanol in solvent (*v*/*v*) and 20 °C) and run 20 (amplitude 35%, 47,5% ethanol in solvent (*v*/*v*) and 35 °C) produced interesting extracts rich in betalains. A previous study performed by Righi Pessoa da Silva et al. [29] about betalain extraction from beetroot by ultrasound bath reported that a maximum of 4.24 mg betacyanins/g of dry sample at an extraction time of 90 min with 25% ethanol in solvent and at 52–37 °C was obtained. In the present study, the maximum obtained content in total betalains was 2-fold higher (11.59 ± 0.11 mg/g dry weight) than the results obtained by these authors for beetroot [29]. In the present study, the extract with the higher betalain content was obtained by a UAE process for 5 min and with a low ethanol volume (%) in the extraction solvent (run 9: amplitude 20%, 15% ethanol in solvent (*v*/*v*) and 60 °C).

In order to analyze the effect of the independent variables in the target responses, an analysis of variance (ANOVA) was conducted (Table S4, Supplementary Materials). The selected quadratic model was significant (*p* < 0.05) to the major betalain responses (extraction yield (%) and content (mg/g dry weight)). Between independent variables, only [ethanol volume (%) in solvent (*v*/*v*)] and [ethanol volume (%) in solvent (*v*/*v*)] ^2^ were significant. These results also agreed with those reported by Melgar et al. [17] and Righi Pessoa da Silva et al. [29], which affirmed that the volume of the organic solvent in the solvent (mixtures methanol/water) had a major influence on betalain extraction. Betalains are hydrophilic pigments; for this reason, in the betalain and polyphenol UAE extraction from beetroot by-products, Fernando et al. [30] concluded that the ethanol/water mixture used as the solvent produced a more efficient extraction yield than the solvent composed only of ethanol (without water). In addition, Cejudo-Bastante et al. [31] reported the effect of pH and temperature on the betalain content and color of the extracts obtained from pitaya peel and concluded that temperature was the main factor for an efficient extraction process, showing a greater effect on the degradation of betalains. In the present study, the applied temperature (°C) and amplitude (%) did not produce any significant effects in the extraction yield and in the content of the betalains in the UAE extracts from *Opuntia stricta* var. *Dillenii* whole fruit

**Figure 2:**
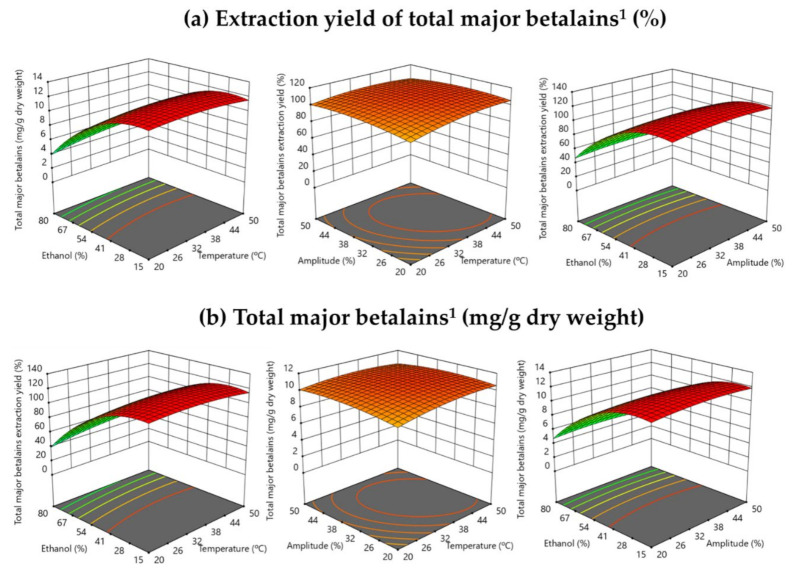
Response surface graph illustrating the effect of the three independent variables (amplitude (%), temperature (°C) and ethanol in solvent (%, *v*/*v*)) on total major betalains (**a**) extraction yield (%) and content (mg/g dry weight). ^1^ The sum of most abundant betalains: betanin, isobetanin, 2′-O-apiosyl-4-O-phyllocactin, 5″-O-E-sinapoyl-2′-apyosil-phyllocactin and neobetanin.

Figure 2 shows the effect of the independent variables on the extraction yield (%) (Figure 2a) and the content of total major betalains (mg/g dry weight) (Figure 2b) by RSM. The ethanol (%) in the extraction solvent was the variable with the strongest effect in the target response (total major betalain content). The higher response was obtained using a lower ethanol volume (%) in solvent (*v*/*v*). These facts could be due to the polarity of betalains, which are water-soluble compounds [32]. Three-dimensional surface graphics also show that the ultrasound amplitude did not have a significant effect on the extraction of betalains (Figure 2), and these results agreed with those reported by Laqui-Vilca et al. [25] in their study of betalain extraction from quinoa. Regarding temperature, this variable did not have a significant effect on the extraction of betalains because betalain compounds are stable until 50–60 °C [28], and the selected range of temperatures in these UAE assays was 20–50 °C (see Section 2), and no betalain degradation was observed.

Betalains are located in the vacuoles within the cytoplasm of parenchyma cells (pulp cells) and also in vesicles in the chlorenchyma (peel cells), as reported in a study about Opuntia spp. fruit microstructure [33]. Ultrasounds formed many small bubbles close to the cell wall which collapse, creating fissures that enabled the extraction of betalains from the vacuoles or vesicles and their solubilization in the extraction solvent.

##### Piscidic Acid

The target responses for piscidic acid were from 109.64 to 249.91% for the extraction yield and 1.02 to 2.32 mg/g dry weight for the total major betalain content, Table 2. The extraction yields using the CDD design combinations were higher than 100%. This means that the present quadratic model of the UAE improved the extraction of piscidic acid compared with the standard extraction by homogenization without ultrasounds (control). This tendency was the same as that observed for the extraction of the major betalains, the proposed model was significant (*p* > 0.05) and ethanol volume (%) in the solvent (*v*/*v*) was the independent variable which influenced the target responses more. The ANOVA data analyses are shown in Table S3 (Supplementary Material).

The lowest extraction yield and piscidic content were obtained at run 3 (amplitude 20%, ethanol 100% in solvent and 35 °C): 109.64 ± 7.65% for extraction yield and 1.02 mg/g dry weight for piscidic content. The highest one also was obtained at run 10 (amplitude 50%, 15% ethanol in solvent (*v*/*v*) and 20 °C): 249.91 ± 0.45% for extraction yield and 2.32 ± 0.08 mg/g dry weight of piscidic acid (Table 2). These results agreed with those reported by Melgar et al. [17] and Righi Pessoa da Silva et al. [30], who concluded that in the ultrasonic extraction of phenolic compounds, a higher ethanol volume in the solvent did not favor the extraction.

**Figure 3:**
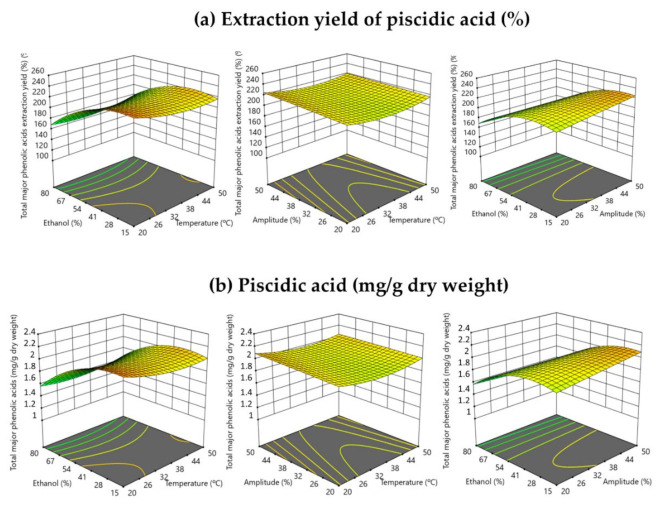
Response surface graph illustrating the effect of the three independent variables (amplitude (%), temperature (°C) and ethanol in solvent (%, *v*/*v*)) on piscidic acid (**a**) extraction yield (%) and content (mg/g dry weight).

Figure 3 shows the 3D response surface graphics of the extraction yield (%) and piscidic acid content (mg/g dry weight) in the UAE assays. In the same way as the previously described results for betalains, it was notorious that only the ethanol volume (%) in the extraction solvent (*v*/*v*) had a significant effect on the piscidic acid content, with a similar trend to what was observed for betalains (Figure 2), although the amplitude (%) and the temperature (°C) of the UAE processes have not shown any significant effect in the extraction of piscidic acid from *Opuntia stricta* var. *Dillenii* whole fruits. A UAE process conducted at a high amplitude (50%) and low temperature (10 °C) produced extracts with a high content of piscidic acid (Table 2). This fact could be related to the greater cavitation forces produced in the extraction mixture when a high-applied amplitude was used, and for this reason more plant material fragmentation was produced. However, in the same way, using high amplitudes, the temperature also increased with the cavitation forces, and this fact could produce the thermal degradation of the phenolic compounds. Therefore, high amplitude with a controlled low temperature, as was carried out in the present study, might increase the extraction yield and the content of piscidic acid in the UAE extracts, but without significant degradation effect (*p* > 0.05).

Gómez-Maqueo et al. [33] in a study about the microstructural analysis of prickly pear tissues submitted to high hydrostatic pressures reported that the piscidic acid was located in chlorenchyma, parenchyma and collenchyma cell walls of the fruit cellular structure. In the present study, the UAE process formed small bubbles that collapse at the cell surface, increasing the pressure, and they could possibly produce the breakdown of the cellular organelles. This reason could explain why the ultrasounds improved the extraction of such phenolics as piscidic acid from *Opuntia stricta* var. *Dillenii* whole fruit using an ethanol/water solvent.

##### Flavonoids

In the case of UAE extraction of flavonoids from *Opuntia stricta* var. *Dillenii* whole fruits, the obtained extracts have 0.07 to 0.40 mg/g dry weight of total major flavonoids, with extraction yields ranging from 21.43% to 121.20% (Table 2). In all obtained UAE extracts, the HPLC profiles of flavonoids were quite similar. As can be seen in Figure S2 (Supplementary Materials), the most abundant flavonoid was isorhammentin glucoxyl-rhamnosyl-pentoside (IG2) (peak 9).

As the above-reported data for UAEextraction of other bioactive compounds from *Opuntia stricta* var. *Dillenii* whole fruits state, the lowest extraction yield of flavonoids was obtained at run 3 (amplitude 20%, ethanol 100% volume in solvent and 35 °C), showing 21.43 ± 2.01% of extraction yield and 0.07 ± 0.00 mg/g dry weight of total major flavonoid content (Table 2). On the other hand, the highest total flavonoid content was obtained at run 8 (amplitude 35%, 47.5% ethanol in solvent (*v*/*v*) and 60 °C) and run 11 (amplitude 35%, 47.5% ethanol in solvent (*v*/*v*) and 35 °C), with 119.57 ± 1.16 and 121.20 ± 1.86% extraction yield, respectively, and a total flavonoid content of 0.39 ± 0.00 and 0.40 ± 0.01 mg/g dry weight, respectively (Table 2). The only difference between these two runs, run 8 and 11, was the process temperature (run 8, 60 °C and run 11, 35 °C). Although the extraction yields at run 8 and run 11 were not significantly different (*p* > 0.05), more flavonoids were extracted at 35 °C than at 60 °C.

These results could indicate that lower temperatures could improve the UAE extraction of flavonoids. However, at run 6 (amplitude 35%, 47.5% ethanol in solvent (*v*/*v*) and a temperature of 10 °C), which had the same amplitude and same ethanol percentage in solvent as runs 8 and 11, the extraction yield (111.37 ± 0.31%) and total flavonoid content (0.36 ± 0.01 mg/g dry weight) were lower. As Pham et al. [34] reported in a study of flavonoid ultrasound-assisted extraction from *Celastrus hindsii* leaves, the process temperature had a positive effect in the extraction of flavonoids up to a point (40 °C), because higher temperatures could have a negative effect. Other previous published studies that compare ultrasound-assisted extraction and Shoxlet extraction from *Opuntia ficus-indica* fruits using methanol/water as a solvent reported that the extraction yield of flavonoids decreased when temperature increased above 43 °C [16]. In the case of the present study, this decrease in total flavonoid content in the UAE extracts took place when the process temperature increased higher than 35 °C. This fact could be because a high temperature causes flavonoid degradation, as we mentioned before for betalain compounds [35]. However, according to ANOVA processing of the obtained data (Table S3, Supplementary Material), only the ethanol volume (%) in extraction solvent (*v*/*v*) had a significant (*p* < 0.05) influence on the extraction yield (%) and on the content of the major flavonoids (mg/g dry weight). For these target responses, the quadratic model was significant (*p* < 0.05).

**Figure 4:**
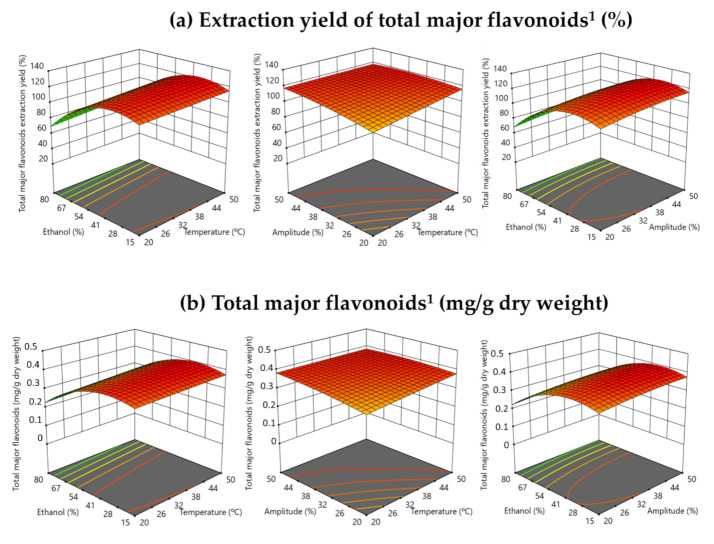
Response surface graph illustrating the effect of the three independent variables (amplitude (%), temperature (°C) and ethanol in solvent (%, *v*/*v*)) on total most abundant flavonoids (**a**) extraction yield (%) and content (mg/g dry weight). ^1^ Sum of major identify flavonoids: Isorhamnentin glucoxyl-rhamnosyl-pentoside (IG2), Quercetin-3-O-rhamnosyl-rutinoside (QG3) and Quercetin glycoside (QG2)—Quercetin hexose pentoside.

Figure 4 shows the effect of the UAE process variables (amplitude (%), temperature (°C) and ethanol volume (%) in solvent (*v*/*v*)) on the total major flavonoid content by RMS graphics. Ethanol volume (%) in the UAE solvent (*v*/*v*) was also the variable with a significant influence in the target responses (recovery yield (%) and total flavonoid content (mg/g dry weight)). These results agreed with the obtained ones in some studies which showed that ethanol volume in the UAE solvent was the most important factor affecting the extraction of flavonoids [34,36].

Flavonoids are also located at the cell walls of *Opuntia* tissues cells (chlorenchyma, and collenchyma (peel cells) and parenchyma (pulp cells)) [33]. Ultrasonic power produced cavitation forces, which broke the plant tissues and cell walls [34]. These fragmentations could cause the release of flavonoids from the cell walls and their passage into the solvent, thus increasing the flavonoid extraction in the same way as for the piscidic acid.

##### In Vitro Biological Activities of UAE Extracts from *Opuntia stricta* var. *Dillenii* Whole Fruits

**Figure 5:**
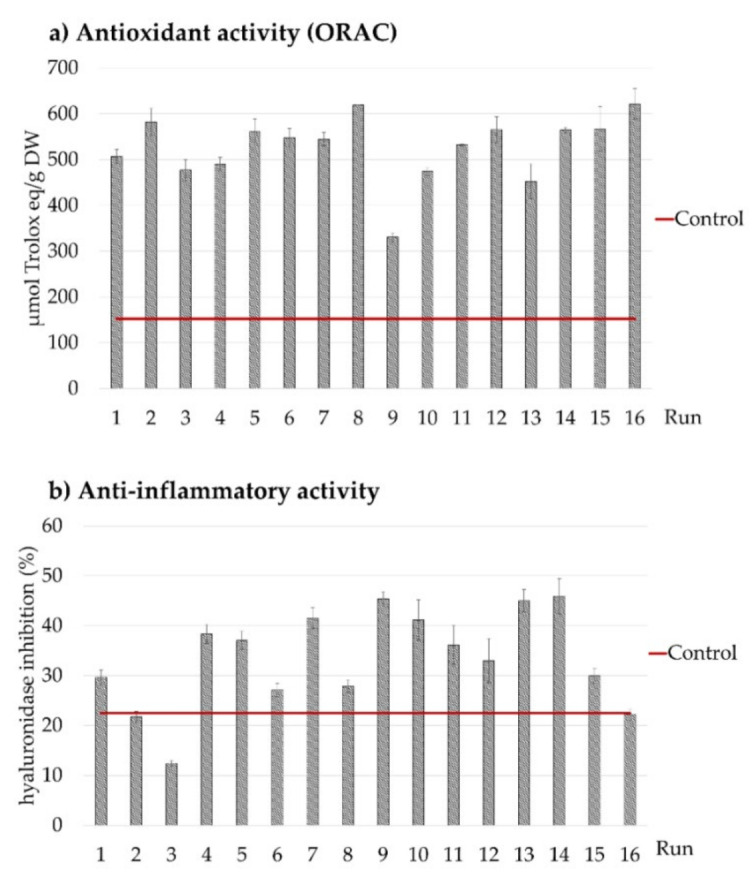
Antioxidant (µmol trolox eq./g dry weigh) and anti-inflammatory activity (% hyalunoronidase inhibition) of the extracts obtained at 5 min UAE time compared to extract obtained with homogenization (control).

Antioxidant activity of UAE extracts was determined by radical absorbance activity, the ORAC method. The lowest antioxidant activity was obtained at run 9 (amplitude 20%, 15% ethanol in solvent (*v*/*v*) and 50 °C), showing a value of 330.71 ± 9.63 µmol trolox eq./g dry weight (Table 2). The higher antioxidant activity was observed in the *Opuntia stricta* var. *Dillenii* extracts obtained by UAE using run 8 (amplitude 35%, 47.5% ethanol in solvent (*v*/*v*) and 60 °C), precisely, 618.85 ± 0.57 µmol trolox eq./g dry weight (Table 2). The proposed model was not significant (*p* > 0.05) in determining the effect of the independent variables on the antioxidant activity of the obtained extracts, because the in vitro assays give us an idea of their capacity, but not of the full biological activity of the extracts, because the same bioactive compound could take part in more than one biological mechanism to give the same biological activity. Nonetheless, it should be noted that all applied combinations of variables in the UAE process gave the same result, and all extracts showed a higher antioxidant capacity than the extracts obtained by standard extraction by homogenization without the use of ultrasounds (Section 2). In Figure 5, the antioxidant activity (ORAC) (a) of all obtained extracts by UAE is shown. The contribution of each bioactive class of compounds on the antioxidant activity of the UAE extracts were significantly different, with flavonoids being the bioactive compounds that showed the highest antioxidant activity with a correlation value (*r* = 0.999) compared to the correlation obtained from antioxidant activity and betalain content (*r* = 0.981) and piscidic acid content (*r* = 0.998). Gómez-Maqueo [24] in a published study of the biological activities (anti-inflammatory and antioxidant) of *Opuntia ficus-indica* L. Mill prickly pears reported that the isorhamnentin glucosides and the piscidic acid contents showed the highest correlations with antioxidant activity (ORAC assay). In the present study, the observed correlations agreed with these results.

Additionally, Figure 5b and Table 2 show the data of the hyaluronidase inhibition capacity (anti-inflammatory activity) of the UAE *Opuntia stricta* var. *Dillenii* whole fruit extracts. The lowest anti-inflammatory activity was observed in extracts obtained at run 3 with 100% ethanol with 12.40 ± 0.62% of hyaluronidase inhibition, and the highest one, 45.91 ± 3.52% of hyaluronidase inhibition activity, was obtained at run 14 with 0% ethanol in solvent (*v*/*v*). The proposed model was not significant (*p* > 0.05) to determine the effect of the independent variables of the anti-inflammatory activity response. Nonetheless, it should be noted that a lower ethanol volume (%) in the UAE extraction solvent mixture rendered a most efficient extraction of bioactive compounds, mainly betalains, pisicidic acid and flavonoids. The contribution of each bioactive class of bioactive compounds on the anti-inflammatory activity of the UAE extracts was significantly different, withthe flavonoids being the *Opuntia stricta* var. *Dillenii* bioactive compounds that showed the highest anti-inflammatory activity with a correlation value (*r* = 0.999) compared to the correlation obtained for the anti-inflammatory activity, betalain content (*r* = 0.981) and piscidic acid content (*r* = 0.998). Gómez-Maqueo [24] concluded that anti-inflammatory activity values (measured by hyaluronidase inhibition) in *Opuntia ficus-indica* L. Mill prickly pears fruit correlated with one of the isorhammentin glycosides, the isorhammentin glucoxyl-rhamnosyl-pentoside (IG2) content (*r* = 0.998). Precisely, the most abundant flavonoid in *Opuntia stricta* var. *Dillenii* whole fruit is the isorhammentin glucoxyl-rhamnosyl-pentoside (IG2), which represents 73.41% of the total identified flavonoids in these fruits. In the present study, flavonoid content in the UAE extracts showed the highest correlation (*r* = 0.999) with anti-inflammatory activity.

**Figure 6:**
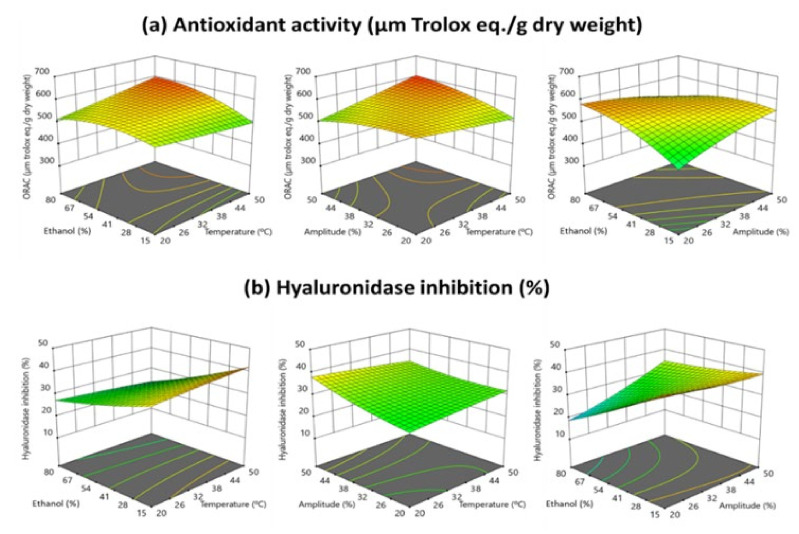
Response surface graph illustrating the quadratic effect of the three independent variables on (**a**) antioxidant activity by ORAC (μmol Trolox eq./g dry weight) (**b**) anti-inflammatory activity by hyaluronidase inhibition (%) of the extracts obtained at 5 min UAE time.

Figure 6 shows the effect of the UAE process variables (amplitude (%), temperature (°C) and ethanol volume (%) in solvent (*v*/*v*)) on the biological activities (antioxidant and anti-inflammatory) of the obtained extracts by RMS graphics. Although the model was not significant, for these responses, it was notorious that the UAE process conducted at a constant temperature (25 °C), at low amplitude (20%) and with a low ethanol volume (%) in solvent (15%, *v*/*v*) produced extracts with higher antioxidant activities (µmol trolox eq./g dry weight) that those obtained by the standard homogenization method of extraction (without ultrasounds). Additionally, this combination (low ethanol volume (%) in solvent (15%, *v*/*v*)) rendered extracts with more anti-inflammatory activities (µmol trolox eq./g dryweight) related to the values of this biological activity in the extracts obtained by the standard extraction process. For both responses (antioxidant and anti-inflammatory activities) this fact might occur because the ethanol volume (%) in the solvent is the variable with the higher influence on the extraction efficacy of flavonoids by UAE and, precisely, these compounds (flavonoids) were the bioactive compounds with a higher correlation with both biological activities: antioxidant and anti-inflammatory activities.

#### 3.2.3. Model Fitting

Using Design-Expert^®^ software, the obtained values of the content of bioactive compounds in the UAE extracts shown in Table 2 were adapted to a polynomial quadratic regression model. Table S3 (Supplementary Materials) shows the ANOVA and regression analysis conducted in the present study. Only the extraction yield Equations (2)–(4) are shown here with their resulting polynomial models. The equations of the models for the bioactive compound contents were quite similar to the data obtained (extraction yields), and the observed influence of the UAE extraction variables was the same. On the other hand, the fitting models for in vitro biological activities (antioxidant and anti-inflammatory) of the UAE extracts were not represented because the models were not significant (*p* > 0.05) as explained before, and the parameter’s combinations cannot be adapted to a polynomial regression model. The coefficients for all responses are available in Table S4 in the Supplementary Materials.

*Y_1_* (*Major betalain extraction yield*) = 57.248 + 1.395X_1_ + 1.680X_2_ + 0.764X_3_ − 0.010X_1_ × X_2_ + 0.009X_1_ × X_3_ − 0.002X_2_ × X_3_ − 0.018X_1_^2^ − 0.017X_2_^2^ − 0.022X_3_^2^ [R^2^ = 0.980; R^2^_Adj_ = 0.949] (2)

*Y_2_* (*Piscidic acid extraction yield*) = 240.038 − 3.289X_1_ +1.101X_2_ + 1.108 X_3_ − 0.005X_1_ × X_2_ + 0.013X_1_ × X_3_ − 0.014X_2_ × X_3_ + 0.039X_1_^2^ − 0.001X_2_^2^ − 0.020X_3_^2^ [R^2^ = 0.879; R^2^_Adj_ = 0.697] (3)

*Y_3_* (*Major flavonoids extraction yield*) = 51.695 + 0.665X_1_ + 1.569X_2_ + 1.251 X_3_ − 0.015X_1_ × X_2_ + 0.003X_1_ × X_3_ − 0.001X_1_^2^ − 0.011X_2_^2^ − 0.020X_3_^2^ [R^2^ = 0.959; R^2^_Adj_ = 0.897] (4)

These mathematical equations illustrate the effect of the independent variables (X_1_: temperature (°C), X_2_: amplitude (%) and X_3_: ethanol in solvent (%, *v*/*v*)) for each response. The parametrical value and each sing represent the expected effect on the response. Negative sing represents an antagonist effect in the response. The main indicators of the model’s significance are R^2^ and R^2^_adj_. The variation around the average explained by the model is represented by R^2^, while R^2^_adj_ results from an adjustment between the significant terms in the model (*p* < 0.05) and the number of variables. Values about 1 illustrate more accordance within experimental and theoretical data [37]. In addition, as the mathematical models have a non-significant lack-of-fit (*p* > 0.05), these indicate that model equations are a good description of the variable effects in the responses.

## 4. Conclusions

UAE processes using green solvents as mixtures of ethanol/water efficiently produce extracts rich in betalains and phenolic compounds, maintaining the original profile (composition) of the starting material, *Opuntia stricta* var. *Dillenii* whole fruit. Between the UAE applied process variables, the ethanol volume (%) in the extraction solvent (*v*/*v*) was the only one with a significant (*p* < 0.05) influence on the extraction yield and on the bioactive content in the extracts. A lower ethanol percentage in the extraction solvent produced a higher content of bioactive compounds in the UAE extract. The best UAE extraction time was 5 min, and the variable combination (run 10, low ethanol volume % in solvent (15%, *v*/*v*), high amplitude (50%) and low temperature (20 °C)) produced the extract with the highest major total betalain content of 10.06 ± 0.10 mg/g dry weight, a piscidic acid content of 2.32 ± 0.08 mg/g dry weight and a major total flavonoid content of 0.38 ± 0.00 mg/g dry weight. The CCD design was not statistically significant (*p* > 0.05) for in vitro biological activities of the obtained UAE extracts (antioxidant and anti-inflammatory). However, all UAE obtained extracts from *Opuntia stricta* var. *Dillenii* whole fruits showed higher bioactivities than the extracts obtained using the conventional method (without ultrasound), and these correlated mainly with the piscidic acid content (antioxidant activity) and with major total flavonoid content (anti-inflammatory activity), and more precisely with the isorhammentin glucoxyl-rhamnosyl-pentoside (IG2) content in the UAE extracts.

Ultrasound-assisted extraction (UAE) with green solvents, such as mixtures of ethanol/water, was a good, innovative, environmentally friendly extraction technology to obtain extracts rich in betalains and phenolic compounds, with an almost unaltered profile of the starting *Opuntia stricta* var. *Dillenii* whole fruits, and with proven in vitro biological activities (antioxidant and anti-inflammatory), for use in the food industry as an ingredient with health benefits. Acoustic cavitation caused by ultrasounds leads to the breakdown of plant cells and allows the bioactive compounds to be efficiently transferred from vacuoles to the green solvent.

## Figures and Tables

**Table 1:** UAE central composite design (CCD) for three independent variables: amplitude (%), ethanol volume % in solvent (*v*/*v*) and temperature (°C).

Variables	Factors	Levels	
		−1	1
Temperature (°C)	X1	20	50
Amplitude (%)	X2	20	50
Ethanol in solvent (%)	X3	15	80
Run number	Factor X1: Temperature (°C)	Factor X2: Amplitude (%)	Factor X3: Ethanol in solvent (%)
1	35	10	47.5
2	20	20	80
3	35	35	100
4	20	20	15
5	50	50	80
6	10	35	47.5
7	50	50	15
8	60	35	47.5
9	50	20	15
10	20	50	15
11	35	35	47.5
12	35	60	47.5
13	20	50	80
14	35	35	0
15	35	35	47.5
16	50	20	80

**Table 2:** UAE Central composite design (CCD) for three independent variables and experimental results of the extraction yield, the content of the most abundant bioactive compounds (total major betalains, total phenolic acids and total major flavonoids) and biological activities (antioxidant and anti-inflammatory) obtained by UAE at 5 min of the central composite design (CCD) design.

Run	Extraction Independet Variables			Extraction Yield % (*w/w*)			Bioactive Compound Content (mg/g Dry Weigth)			In Vitro Biological Activities	
	Amplitude (%)	EtOH in Solvent (%, *v*/*v*)	Temperatura (°C)	Total Major Betalains ^1^	Total Phenolic Acids ^2^	Total Major Flavonoids ^3^	Total Major Betalains ^1^	Total Phenolic Acids ^2^	Total Major Flavonoids ^3^	Antioxidant Activity. (µmol Trolox eq./g Dry Weight)	Hyaluronidase Inhibition (%)
**1**	10	47.5	35	102.91 ± 0.91 ^d^	197.13 ± 3.80 ^d^	106.91 ± 2.10 ^de^	10.34 ± 0.09 ^d^	1.84 ± 0.09 ^d^	0.35 ± 0.01 ^de^	506.84 ± 15.01 ^cd^	29.66 ± 1.48 ^b^
**2**	20	80	20	29.42 ± 1.36 ^b^	181.03 ± 0.52 ^c^	62.05 ± 4.03 ^b^	2.96 ± 0.14 ^b^	1.68 ± 0.10 ^c^	0.20 ± 0.02 ^b^	582.07 ± 29.35 ^fg^	21.72 ± 1.09 ^ab^
**3**	35	100	35	0.43 ± 0.08 ^a^	109.64 ± 7.65 ^a^	21.43 ± 2.01 ^a^	0.04 ± 0.01 ^a^	1.02 ± 0.07 ^a^	0.07 ± 0.00 ^a^	477.05 ± 22.94 ^bc^	12.40 ± 0.62 ^a^
**4**	20	15	20	102.38 ± 8.99 ^d^	222.29 ± 5.95 ^de^	92.46 ± 8.08 ^cd^	10.29 ± 0.90 ^d^	2.07 ± 0.05 ^e^	0.30 ± 0.03 ^cd^	489.84 ± 14.70 ^bc^	38.37 ± 1.92 ^c^
**5**	50	80	50	47.29 ± 4.95 ^c^	180.77 ± 4.39 ^c^	87.09 ± 7.14 ^c^	4.75 ± 0.50 ^c^	1.68 ± 0.04 ^c^	0.28 ± 0.02 ^c^	560.80 ± 28.04 ^e^	37.03 ± 1.85 ^c^
**6**	35	47.5	10	100.69 ± 2.00 ^d^	231.92 ± 3.95 ^de^	111.37 ± 0.31 ^e^	10.12 ± 0.12 ^d^	2.16 ± 0.03 ^e^	0.36 ± 0.00 ^de^	548.11 ± 20.08 ^de^	27.15 ± 1.36 ^b^
**7**	50	15	50	106.60 ± 1.26 ^d^	224.53 ± 2.06 ^de^	111.30 ± 4.42 ^e^	10.71 ± 0.01 ^d^	2.09 ± 0.02 ^e^	0.36 ± 0.01 ^de^	544.75 ± 14.37 ^d^	41.53 ± 2.08 ^d^
**8**	35	47.5	60	105.12 ± 1.99 ^d^	227.48 ± 8.01 ^de^	119.57 ± 1.16 ^e^	10.57 ± 0.19 ^d^	2.12 ± 0.07 ^e^	0.39 ± 0.00 ^de^	618.85 ± 0.57 ^g^	27.86 ± 1.29 ^b^
**9**	20	15	50	111.34 ± 9.72 ^d^	217.91 ± 14.58 ^de^	109.42 ± 4.38 ^e^	11.59 ± 0.11 ^d^	2.03 ± 0.14 ^e^	0.36 ± 0.01 ^de^	330.71 ± 9.63 ^a^	45.43 ± 1.41 ^e^
**10**	50	15	20	115.29 ± 3.05 ^d^	249.91 ± 0.45 ^e^	116.83 ± 0.80 ^e^	11.06 ± 0.10 ^d^	2.32 ± 0.08 ^e^	0.38 ± 0.00 ^de^	473.93 ± 8.06 ^bc^	41.17 ± 4.97 ^d^
**11**	35	47.5	35	110.05 ± 1.45 ^d^	242.28 ± 5.70 ^de^	121.20 ± 1.86 ^e^	11.43 ± 0.23 ^d^	2.25 ± 0.05 ^e^	0.40 ± 0.01 ^de^	532.20 ± 1.01 ^d^	36.17 ± 3.94 ^c^
**12**	60	47.5	35	103.77 ± 1.95 ^d^	211.91 ± 3.10 ^de^	111.24 ± 2.07 ^e^	10.43 ± 0.03 ^d^	1.97 ± 0.19 ^de^	0.36 ± 0.01 ^de^	565.44 ± 28.40 ^e^	33.01 ± 4.44 ^bc^
**13**	50	80	20	30.10 ± 0.12 ^b^	164.50 ± 4.38 ^b^	77.20 ± 0.67 ^bc^	3.03 ± 0.01 ^b^	1.53 ± 0.00 ^b^	0.25 ± 0.00 ^bc^	452.26 ± 37.94 ^b^	45.00 ± 2.25 ^e^
**14**	35	0	35	114.78 ± 3.65 ^d^	196.59 ± 7.26 ^d^	106.23 ± 6.31 ^de^	11.54 ± 0.37 ^d^	1.87 ± 0.07 ^d^	0.35 ± 0.02 ^de^	564.44 ± 6.18 ^e^	45.91 ± 3.52 ^e^
**15**	35	47.5	35	102.89 ± 4.07 ^d^	184.92 ± 11.92 ^c^	108.86 ± 11.07 ^e^	10.34 ± 0.41 ^d^	1.72 ± 0.11 ^c^	0.36 ± 0.04 ^de^	566.85 ± 48.85 ^e^	29.98 ± 4.04 ^b^
**16**	20	80	50	47.15 ± 5.24 ^c^	185.96 ± 4.97 ^c^	76.81 ± 0.61 ^bc^	4.74 ± 0.53 ^c^	1.73 ± 0.05 ^c^	0.25 ± 0.00 ^bc^	621.58 ± 33.03 ^f^	22.24 ± 8.38 ^ab^

Results were expressed as mean ± standard deviation (*n* = 4). Superscript letters indicate statistically significant differences (*p* ≤ 0.05) between the different conditions of the appliedEAU process. This came from obtaining at least two independent extracts (*n* = 2) and performing the HPLC determinations of each two times (*n* = 2). Superscript small letters indicate statistically significant differences (*p* ≤ 0.05) between CCD runs. ^1^ Sum of most abundant betalains: betanin, isobetanin, 2′-O-apiosyl-4-O-phyllocactin, 5″-O-E-sinapoyl-2′-apyosil-phyllocactin and neobetanin. ^2^ Major phenolic acid: pisicidic acid. ^3^ Sum of the most abundant flavonoids: Isorhamnetin glucoxyl-rhamnosyl-pentoside (IG2). Quercetin-3-O-rhamnosyl-rutinoside (QG3) and Quercetin glycoside (QG2)—Quercetin hexose pentoside.

## Data Availability

Data is contained within the article and Supplementary Material.

## Supplementary Materials

The following are available online at https://www.mdpi.com/article/10.3390/antiox10111786/s1, Figure S1: HPLC-DAD chromatogram of major betalains and phenolic compounds in *Opuntia stricta* var. *Dilleniiat* at 280, 370, 480, and 535 nm. Numbers correspond to the identified compounds indicated at Supplementary Table S2, Figure S2: HPLC-DAD chromatograms obtained at 280 nm, 370 nm, 480 nm and 535 nm of the obtained extract from ultrasound assisted extraction (UAE) at 50% amplitude, 15% ethanol in solvent (ethanol/water, 15/85, *v*/*v*) and 20 °C temperature parameter combination (run 10). Numbers correspond to the identified compounds indicated at Supplementary Table S2, Table S1: Physico-chemical analysis, total betalain and phenolic compound content and in vitro biological activities (antioxidant and anti-inflammatory of *Opuntia stricta* var. *Dillenii*’s prickly pears from Canary Island, Table S2: Individual betalain and phenolic compound content (mg/g dry weight) of *Opuntia strictavar*. *Dillenii*’s whole fruit from Canary Island, Table S3: Major bioactive compounds of *Opuntia stricta* var. *Dillenii* chromatographic identification (retention time (Rt), maximum absorption (λmax), masa spectra according to Gomez-Lopez et al., (2021), Table S4: Analysis of variance and model fitting regression coefficient of independent variables (ethanol volume (%) in solvent (*v*/*v*), amplitude (%) and temperature (°C)) about the extraction yield (%) and content (mg/g dry weight) of most abundant betalains, piscidic acid and flavonoids obtained at 5 min UAE time.
