# Supplementary material for: Ultrasound-Assisted “Green” Extraction (UAE) of Antioxidant Compounds (Betalains and Phenolics) from Opuntia stricta var. Dilenii’s Fruits: Optimization and Biological Activities
Source: Antioxidants (Basel). 2021 Nov 8;10(11):1786. doi: 10.3390/antiox10111786 (PMC8614683; doi:10.3390/antiox10111786)
Supplement: Supplementary file 1 [file antioxidants-10-01786-s001.zip › antioxidants-1423555-supplementary.pdf]

Supplementary Table S1. Physico-chemical analysis, total betalain and phenolic compound content and in vitro biological activities (antioxidant and anti-inflammatory of *Opuntia stricta* var. *Dillenii*'s prickly pears from Canary Island.

**Analysis**	***Opuntia stricta* var. *Dillenii* whole fruit**
Physicochemical características	
Soluble solids (ºBrix)	12.63 ± 0.85
pH	3.32 ± 0.02
Tiritable acidity (%)	1.58 ± 0.10
Wet basis moisture (%)	19.75 ± 1.92
Color (CIELab)	
*L (lighteness)**	30.9197 ± 1.389
*a (greennes-redness)**	4.477 ± 0.3260
*b (blueness-yellowness)**	(-7.456) ± 0.506
Total bioactive content (mg/ g dry weight)	
Total major betalains	10.05 ± 0.08
Total Pisicidic acid	0.93 ± 0.00
Total major flavonoids	0.33 ± 0.00
In vitro Biological activities	
Antioxidant activity (ORAC) (μmol Trolox eq./g dry weight)	151.81 ± 1.86
Hialuronidase inhibition (%)	22.51 ± 2.52

Supplementary Table S2. Individual betalain and phenolic compound content (mg/g dry weight) of *Opuntia stricta* var. *Dillenii*'s whole fruit from Canary Island.

**Bioactive content (mg/g dry weight)**	***Opuntia stricta* var. *Dillenii* whole fruit**
Major betalains	
Betanin	2.74 ± 0.02
Isobetanin	1.68 ± 0.01
2´-O-apiosyl-4-O-phyllocactin	1.22 ± 0.01
5´´-O-E-sinapoyl-2´-apyosil-phyllocactin	2.77 ± 0.01
Neobetanina	1.64 ± 0.00
Total major betalains	10.05 ± 0.08
Phenolic acid	
Pisicidic acid	0.93 ± 0.00
Major Flavonoids	
Quercetin-3-O-rhamnosyl-rutinoside (QG3)	0.02 ± 0.01
Quercetin hexose pentoside (QG2)	0.05 ± 0.00
Isorhamnetin glucoxyl-rhamnosyl-pentoside (IG2)	0.26 ± 0.01
Total major flavonoids	0.33 ± 0.00

All characterization was done on a previous study (Gomez-Lopez et al; 2021)

Supplementary Table S3. Major bioactive compounds of *Opuntia stricta* var. *Dillenii* chromatographic identification (retention time (Rt), maximum absorption (λmax), masa spectra according to Gomez-Lopez et al., (2021).

Peak*	t_R (min)	Compounds	UV λmax (nm)	$[M-H]^+$	$[M-H]^-$	MS/MS (*m/z*)
		**Phenolic acid**				
1	8.150	Piscidic acid	272		255	193, 165, 135, 119 107
		**Major betalains**				
2	10.764	Betanin	535	551		390, 389
3	15.54	Isobetanin	535	551		390, 389
4	27.877	2´-O-apiosyl-4-O-phyllocactin	537		767	551
5	29.281	5´´-O-*E*-sinapoyl-2´-apyosil-phyllocactin	248,330,540		975	---
6	30.784	Neobetanin	467		549	387
		**Major flavonoids**				
7	34.790	Quercetin-3-O-rhamnosyl-rutinoside (QG3)	358	757		611, 303
8	39.756	Quercetin glycoside(QG2) - Quercetin hexose pentoside	255, 353	653		303, 177
9	42.271	Isorhamnetin glucoxyl-rhamnosyl-pentoside(IG2)	254, 356	757		317, 167, 86

All characterization was done on a previous study (Gomez-Lopez et al; 2021)

* Peak numbers are according to Figure S1

Table S4. Analysis of variance and model fitting regression coefficient of independent variables (ethanol volume (%) in solvent (*v/v*), amplitude (%) and temperature (°C)) about the extraction yield (%) and content (mg/g dry weight) of most abundant betalains, piscidic acid and flavonoids obtained at 5 minutes UAE time.

**Extraction yield (%)**									
	**Major betalains[1]**			**Piscidic acid**			**Major flavonoids[2]**		
**Statistical data**	**Regression coefficient**	**F-value**	**p-value**	**Regression coefficient**	**F-value**	**p-value**	**Regression coefficient**	**F-value**	**p-value**
**Model**	57.248	31.935	0.000*	240.038	4.832	0.034*	51.695	15.537	0.002*
X_1-Temperature	1.395	1.817	0.226	-3.289	0.056	0.821	0.655	2.554	0.161
X_2-Amplitude	1.680	0.100	0.762	1.101	0.269	0.623	1.569	3.547	0.109
X_3-Ethanol % in solvent	0.764	204.785	0.000*	1.108	22.104	0.003*	1.251	63.555	0.000*
X_1*X_2	-0.010	0.562	0.482	-0.005	0.027	0.874	-0.015	1.309	0.296
X_1*X_3	0.009	2.039	0.203	0.013	0.907	0.378	0.003	0.306	0.600
X_2*X_3	-0.002	0.092	0.772	-0.014	1.038	0.348	0.000	0.001	0.974
X_1^2	-0.018	2.071	0.200	0.039	1.998	0.207	-0.001	0.002	0.965
X_2^2	-0.017	1.886	0.219	-0.001	0.001	0.971	-0.011	0.754	0.419
X_3^2	-0.022	52.588	0.000*	-0.020	8.451	0.027*	-0.020	47.779	0.000*
Lack of Fit		3.243	0.397		0.068	0.988		0.927	0.653
R^2	0.980			0.879			0.959		
Adj R^2	0.949			0.697			0.897		
Adecuate precission	18.384			8.350			13.894		
**Bioactive content (mg/g dry weight)**									
	**Major betalains[1]**			**Piscidic acid**			**Major flavonoids[2]**		
**Statistical data**	**Regression coefficient**	**F-value**	**p-value**	**Regression coefficient**	**F-value**	**p-value**	**Regression coefficient**	**F-value**	**p-value**
**Model**	5.754	31.940	0.0002*	2.232	4.830	0.0343*	0.169	15.540	0.0017*
X_1-Temperature	0.140	1.820	0.226	-0.031	0.056	0.821	0.002	2.550	0.161
X_2-Amplitude	0.169	0.100	0.763	0.010	0.269	0.623	0.005	3.550	0.109
X_3-Ethanol % in solvent	0.077	204.790	< 0.0001**	0.010	22.100	0.0033*	0.004	63.560	0.0002**
X_1*X_2	-0.001	0.562	0.482	0.000	0.027	0.874	0.000	1.310	0.296
X_1*X_3	0.001	2.040	0.203	0.000	0.907	0.378	0.000	0.306	0.600
X_2*X_3	0.000	0.092	0.773	0.000	1.040	0.348	0.000	0.001	0.974
X_1^2	-0.002	2.070	0.200	0.000	2.000	0.207	0.000	0.002	0.965
X_2^2	-0.002	1.890	0.219	0.000	0.002	0.971	0.000	0.754	0.419
X_3^2	-0.002	52.590	0.0003*	0.000	8.450	0.0271*	0.000	47.780	0.0005**
Lack of Fit		3.240	0.397		0.068	0.988		0.927	0.653
R^2	0.980			0.879			0.959		
Adj R^2	0.949			0.697			0.897		
Adecuate precission	18.384			8.350			13.894		

* Significant

[1] Sum of major identified betalains: Betanin, Isobetanin, 2´-O-apiosyl-4-O-phyllocactin, 5´´-O-E-sinapoyl-2´-apyosil-phyllocactin and Neobetanin

[2] Major phenolic acid identify: pisicidic acid

[3] Sum of major identify flavonoids: Isorhamnetin glucoxyl-rhamnosyl-pentoside (IG2), Quercetin-3-O-rhamnosyl-rutinoside (QG3) and Quercetin glycoside (QG2) - Quercetin hexose pentoside

R^2: coefficient of determination

Adj R^2: adjusted coefficient of determination

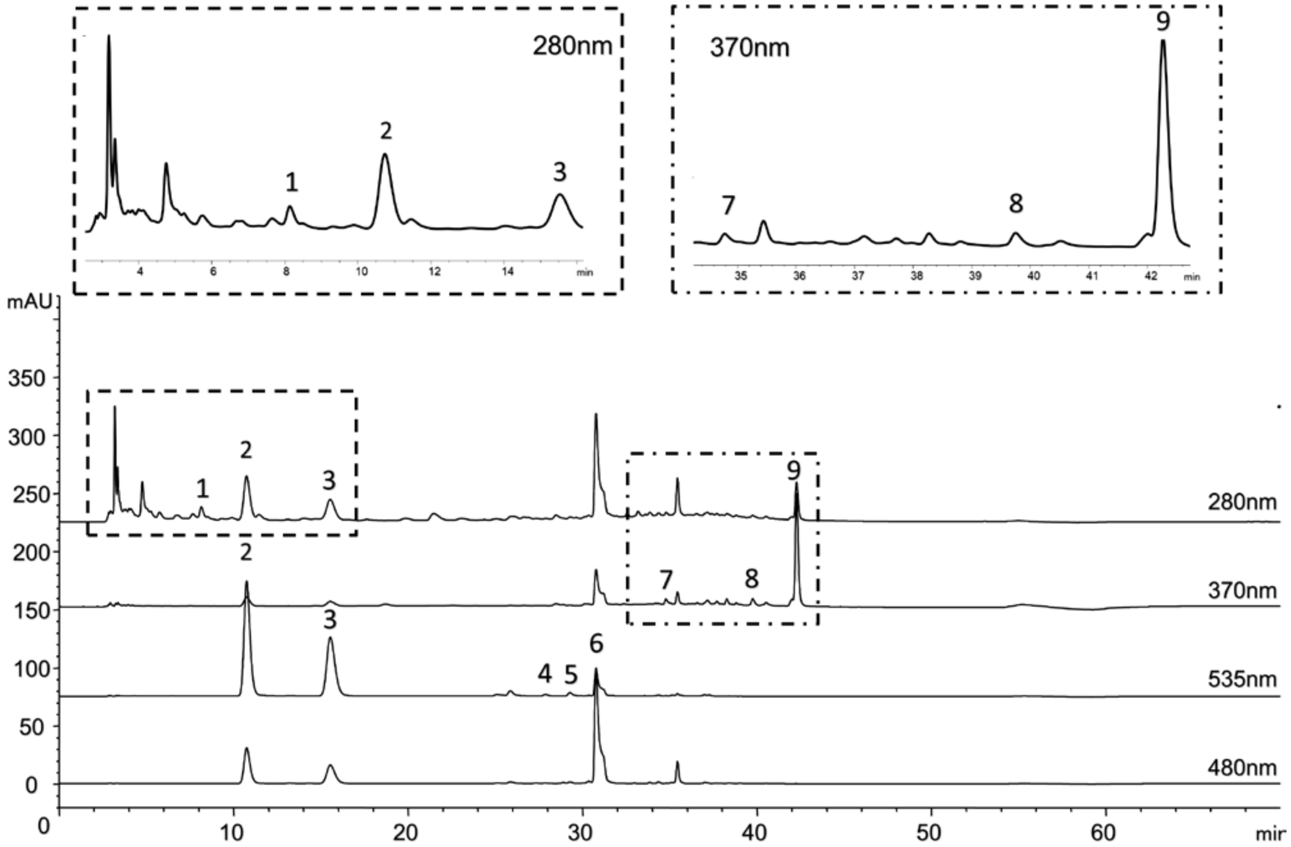

Supplementary Figure S1. HPLC-DAD chromatogram of major betalains and phenolic compounds in *Opuntia stricta* var. *Dillenii* at 280, 370, 480, and 535nm. Numbers correspond to the identified compounds indicated at Supplementary Table S2.

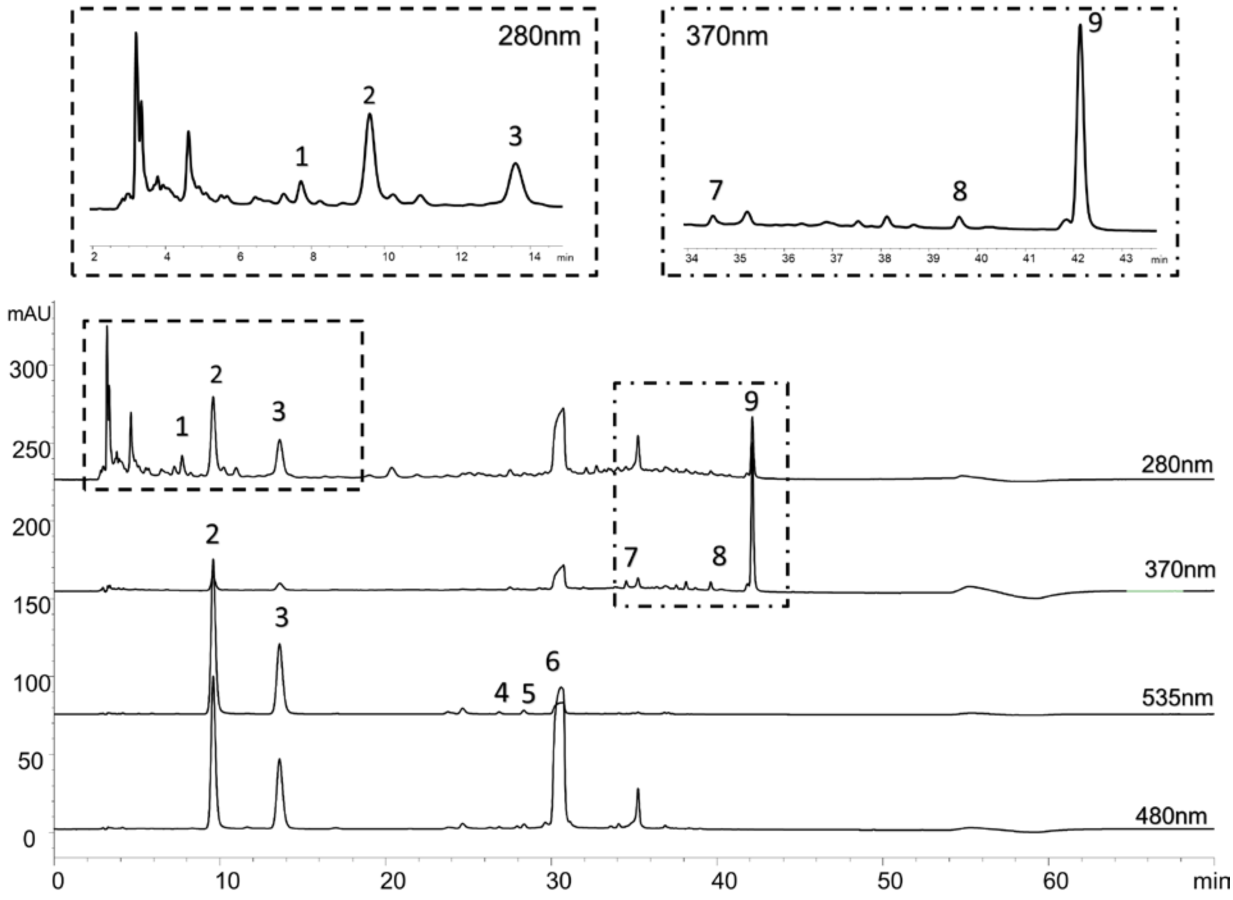

Supplementary Figure S2. HPLC-DAD chromatograms obtained at 280nm, 370nm, 480nm and 535nm of the obtained extract from ultrasound assisted extraction (UAE) at 50% amplitude, 15% ethanol in solvent (ethanol/water, 15/85, *v/v*) and 20°C temperature parameter combination (run 10). Numbers correspond to the identified compounds indicated at Supplementary Table S2.
